# Hypertension prevalence, awareness, treatment and control in Ghanaian population: Evidence from the Ghana demographic and health survey

**DOI:** 10.1371/journal.pone.0205985

**Published:** 2018-11-07

**Authors:** Olutobi Adekunle Sanuade, Sandra Boatemaa, Mawuli Komla Kushitor

**Affiliations:** 1 Institute of Advanced Studies, University College London (UCL), London, United Kingdom; 2 Centre for Complex Systems in Transitions, Stellenbosch University, Stellenbocsh, South Africa; 3 Regional Institute for Population Studies, University of Ghana, Accra, Ghana; Loyola University Chicago, UNITED STATES

## Abstract

Hypertension is a major cause of cardiovascular disease morbidity and mortality in Ghana. This study examines the prevalence, awareness, treatment and control of hypertension among Ghanaian aged 15–49 years. This cross-sectional study retrieved data from the 2014 Ghana Demographic and Health Survey (GDHS). The sample, comprising of 13,247 respondents aged 15–49 years, was analysed using descriptive statistics, Chi-Square tests, independent sample t-tests and binary logistic regressions. The overall prevalence of hypertension was 13.0% (12.1% for males and 13.4% for females). Among respondents who had hypertension, 45.6% were aware of their hypertension status; 40.5% were treating the condition while 23.8% had their blood pressure controlled (BP <140/90 mmHg). Socio-economic and demographic factors, health insurance coverage and recent visit to health facilities played significant roles in hypertension prevalence and awareness. While region of residence and health facility visits were predictors of hypertension treatment, age and region of residence predicted hypertension control in this population. This study suggests that in order to address the increasing burden of hypertension in Ghana, there should be an expansion of the National Health Insurance Scheme and development of measures to reduce health inequities. Also, some of the determining factors such as age, gender, marital status are similar to other cultures; therefore, existing interventions from those cultures could be adapted in addressing hypertension prevalence, awareness, treatment and control in Ghana.

## Introduction

Hypertension is a global public health issue and it contributes significantly to cardiovascular disease, kidney failure, premature deaths and disabilities [1]. Recent evidence shows that between 1990 and 2015, there has been an increase in hypertension incidence, prevalence and deaths globally [2]. In Ghana, population-based studies have shown increase in hypertension prevalence and its significant impact on stroke morbidity and mortality, over the last four decades [3–5]. Despite this, hypertension awareness, treatment and control are poor in the country [6,7].

The rise in the burden of hypertension has been attributed to high population growth especially in urban areas, increase in life expectancy and lifestyle factors [8]. In a review of population based studies on hypertension in Ghana, researchers observed that obesity, increases in waist-to-hip circumference, harmful use of alcohol, lack of exercise and poor diets were associated with the rising prevalence of hypertension [9]. The risk factors of hypertension have been reported to increase across many African countries resulting in the high burden of the condition [10]. Besides human factors, a weak health care system has been implicated in the poor management of hypertension. Increasing morbidity and mortality from hypertension and its complications reflect the inability of the health care system to promptly detect and management hypertension within the general population [11,12].

Ghana’s weak health care system is characterised by limited funding, uncoordinated care, shortage of qualified health workforce, low insurance coverage and inconsistent supply of medication [13–15]. Poor accessibility to health is further compounded by unequal geographic distribution of limited health workforce where urban centres have greater access to the health labour force [16]. In addition, research shows that about 80% of the health care budget is allocated to infectious diseases at the expense of the rising burden of cardiovascular diseases such as hypertension [15,16]. While the National Health Insurance Scheme (NHIS) was instituted as a broad systems level social strategy to reduce the cost of health and to protect vulnerable people from catastrophic health financing, research still shows that about 30% of Ghanaians are not covered by the insurance scheme [17]. This suggests that although some level of hypertension treatment is covered by the insurance, some people may not actually benefit from the insurance coverage. Besides these challenges associated with the formal healthcare system in Ghana, traditional and faith-based healers complicate the management of hypertension by providing unregulated care [11,18]. A pluralistic medical environment, characterised by the provision of healthcare by multiple and competing health systems, creates a situation where individuals healer shop [18,19]. Healer shopping affects continuity of care and results in the poor control of chronic diseases in many parts of Africa [20,21]. Given all these challenges, several studies have reported a poor control of hypertension leading to high morbidity and mortality from a potentially preventable condition [6,10,22,23].

Due to the increase in the burden of hypertension in Ghana, and the visible impact on cardiovascular disease [8], the Government of Ghana, through the joint effort of the Ghana Statistical Service (GSS), the Ghana Health Service (GHS), and the National Public Health Reference Laboratory (NPHRL), decided to collect specialised data on blood pressure in the 2014 Ghana Demographic and Health Survey (GDHS) [24]. This data is the first of its kind in the history of population and health surveys in Ghana. The focus of this data was to monitor the hypertension status of Ghanaians and to come up with intervention strategies that can reduce the burden of the condition in the country [24]. This study examines the prevalence, awareness, treatment and control of hypertension among Ghanaian’s aged 15–49 years. The information that will be generated from this study will be useful for policy decisions and for planning, monitoring and evaluating programmes related to the general health of the Ghanaian population.

## Materials and methods

### Data

This cross-sectional study retrieved data from the 2014 Ghana Demographic and Health Survey (GDHS). Although a total of 13,265 respondents (3,869 males and 9,396 females) aged 15–49 years were approached for blood pressure measurements during the survey, 18 respondents did not have information on their blood pressure readings and they were excluded in our analysis. The sample used for this study was 13,247 respondents with complete information on their blood pressure measurements. The 2014 Ghana Demographic and Health Survey (GDHS) was carried out by the Ghana Statistical Service (GSS), the Ghana Health Service (GHS), and the National Public Health Reference Laboratory (NPHRL) of the GHS. The survey was designed to assist policymakers and programme managers in evaluating and designing programmes and strategies for improving the health of Ghanaians. Ethical clearance was provided by the Ghana Health Service Ethical Review Committee, Research and Development Division, Ghana Health Service; and the Institutional Review Board of ICF International. The Demographic and Health Survey (DHS) team anonymized all data before making them available online. Ghana Statistical Service did not take data from participants’ medical records, rather, anthropometric and blood pressure measurements were taken as part of the data collection [24]. Respondents provided written informed consent before participation in the study.

The 2014 GDHS adopted a two-stage sampling strategy to allow estimates of key indicators at the national level, urban and rural areas and each of the country’s administrative regions [24]. The first stage of the sample design involved selection of clusters consisting of enumeration areas (EAs) delineated for the 2010 Ghana Population and Housing Census (PHC). As a result, a total of 427 clusters were selected in the entire country (216 clusters in urban areas and 211 clusters in rural areas). The second stage involved household listing in all the selected EAs. About 30 households were selected from each cluster through a systematic random sampling, and a total of 12,831 households were selected throughout the country. Hence, all women age 15–49 and men age 15–59 who were either permanent residents of the selected households or visitors who stayed in the household the night before the survey were eligible to be interviewed. However, while blood pressure measurement was taken for all the selected households for the female survey, it was only taken for half of the households selected for the male survey [24]. A response rate of 99% of all the selected households was achieved during the survey. With respect to eligible individuals interviewed, 97% of the eligible women and 95% of eligible men were interviewed [24].

### Measurements

Three blood pressure measurements were taken from consenting participants in all the selected households for the survey. Blood pressure was measured using the LIFE SOURCE UA-767 Plus blood pressure monitor and measurements were taken at intervals of 10 minutes or more [24]. In these analyses, hypertension was defined as an average systolic blood pressure (SBP) ≥ 140 mmHg and/or an average diastolic blood pressure (DBP) ≥90 mmHg of the last two of the three blood pressure measurements taken [25]. Hypertension awareness was defined as self-report of previous diagnosis by a health professional. Treatment of hypertension was defined as self-reported use of any of the followings for management of hypertension: use of anti-hypertensive medicines; weight control; reduction in salt intake; exercise; reduction in alcohol intake or; smoking cessation, according to the Ghana Health Service treatment guidelines [26]. Hypertension control was defined as an average systolic BP of <140 mmHg and an average diastolic BP of <90 mmHg of the last two of the three blood pressure measurements.

The covariates that were examined include: sex (male, female); age (15–24 years, 25–34 years, 35–44 years, 45–49 years); marital status (never married, currently married, formerly married); level of education (no education, primary/junior high school, secondary/higher); place of residence (rural, urban), region of residence (Western, Central, Greater Accra, Volta, Eastern, Ashanti, Brong Ahafo, Northern, Upper East, Upper West); ethnicity (Akan, Ga Adangbe, Ewe, Mole-Dagbani and other ethnic groups), smoking status (non-smokers, smokers), National Health Insurance coverage (no, yes), visit to health facilities in the last 6 months (no visit, hypertension related visit and non-hypertension related visit), and frequency of fruits and vegetable consumption, wealth quintiles and occupation.

Wealth quintiles were classified into poorest, poorer, middle, richer, and richest. The wealth index was constructed from household asset data using principal components analysis. These assets consisted of a television, bicycle, or car, as well as dwelling characteristics such as a source of drinking water, sanitation facilities, and type of flooring material [26]. After computing the index, national-level wealth quintiles were obtained by assigning the household score to each de jure household member, ranking each person in the population by his or her score, and then dividing the ranking into five equal categories (from poorest to richest). Occupation was classified into four levels. Level 1 included those who were unemployed (e.g., Students). Level 2 activities were defined as sedentary work, mostly done while sitting (e.g., Driving, business and administrative work); level 3 activities involved standing or occasional walking (e.g., Teaching, sales related work), and; level 4 activities involved those causing a significant increase in heart rate and sweating, and were usually performed outdoors (e.g., Farming, construction work) [27].

### Data analysis

Mean and standard deviations were used to describe continuous variables and percentages were used to describe categorical variables. Cross tabulations and Chi-Square tests were used to describe the variations in hypertension prevalence, awareness, treatment and control by the covariates that were categorical. Independent sample t-tests were used to show the association between frequency of fruit and vegetable consumptions and hypertension prevalence, awareness, treatment and control. Binary logistic regression was used to examine the correlates of hypertension prevalence, awareness, treatment and control. Appropriate survey weights were applied before the analysis. Since the 2014 GDHS used a two-stage stratified cluster sampling technique, sampling weight was calculated based on sampling probabilities separately for each sampling stage and for each cluster [26]. During the analysis, the first thing that we did was to generate a weight variable using the sampling weight. After this, we set up the survey weight in STATA using the generated weight, primary sampling unit and strata. This was then applied in all the analysis. We also tested for multicollinearity using variance inflation factor (VIF) and tolerance (S1 Table). As a general rule of thumb, a variable with VIF values greater than 10 or tolerance value of 0.1 and above indicates multicollinearity and imply further investigation. From the output, all the VIF values were less than 10. The data were analysed using STATA 12.

## Results

### Characteristics of the participants

The characteristics of the population are shown in Table 1. A total of 13,247 respondents were included in the analysis and there were more women (70.9%) than men (29.1%). The mean age of the respondents was 29.6 (± 9.8) years and a higher proportion was currently married (55%). The majority of the sample had basic education (i.e. primary and JHS) and the highest proportion (40.5%) engaged in level 1 occupation (vigorous intensity related work). With respect to wealth status, the highest proportion was in the poorer and poorest wealth quintile (43.4%). Of the ten regions, Western Region had the highest proportion of respondents; slightly more than half (51.1%) of the sample lived in rural areas. More than four out of ten (41.0%) were Akan, 11.8% were Ewe and about 17.0% belonged to other ethnic groups such as Mande, Guan, Grusi, Gurma, etc.

**Table 1:** Background characteristics.

	Number (13247)	Percentage
**Sex**		
Male	3855	29.1
Female	9392	70.9
**Age**		
15–24	4835	36.5
25–34	3979	30.0
35–44	3205	24.2
45–49	1228	9.3
**Marital status**		
Never married	4895	37.0
Currently married	7288	55.0
Formerly married	1064	8.0
**Level of education**		
No education	2781	21.0
Primary/JHS	7421	56.0
Secondary and above	3045	23.0
**Occupational physical activities**		
Unemployed	2995	22.6
Sedentary work	439	3.3
Moderate intense work	4448	33.6
Vigorous intense work	5365	40.5
**Wealth status**		
Poorest	3324	25.1
Poorer	2475	18.7
Middle	2636	19.9
Richer	2496	18.8
Richest	2316	17.5
**Place of residence**		
Rural	6820	51.5
Urban	6427	48.5
**Region**		
Western	1474	11.1
Central	1304	9.8
Greater Accra	1421	10.7
Volta	1107	8.4
Eastern	1283	9.7
Ashanti	1429	10.8
Brong Ahafo	1425	10.8
Northern	1473	11.1
Upper East	1296	9.8
Upper West	1035	7.8
**Ethnicity**		
Akan	5432	41.0
Ga Adangbe	747	5.6
Ewe	1568	11.8
Mole-Dagbani	3200	24.2
Others	2300	17.4
**Hypertension categories**		
Normotensive	8608	65.0
Pre-hypertensive	2916	22.0
Hypertensive	1723	13.0
**Smoking**		
Non-smokers	12966	97.9
Smokers	281	2.1
**NHIS coverage**		
No	5069	38.3
Yes	8178	61.7
**Visited to health facility in the last 6 months**		
No visit	9026	68.2
Hypertension related	97.0	0.7
Non-hypertension related	4124	31.1
**Frequency of taking vegetables in a week**		
***Fruit consumption (mean*, *days)***	13247	2.5 (± 1.5)
***Vegetable consumption (mean*, *days***	13247	2.8 (± 1.4)

JHS- Junior High School

With regard to blood pressure categories, 65.0% were normotensive, 22.0% were pre-hypertensives and 13.0% were hypertensive. A small proportion of the respondents (2.1%) were smokers and 61.7% was covered by the National Health Insurance Scheme (NHIS). With respect to visit to a health facility, while about two-thirds (68.2%) did not visit the health facility in the last 6 months prior to the survey, 0.7% visited for hypertension related reason and 31.1% visited for non-hypertension related reason. The mean number of days that respondents consumed fruits and vegetables in a week was 2.5 days and 2.8 days, respectively. When those who were hypertensives were asked what specific actions they were taking to lower their blood pressure, 32.6% were taking prescribed medication, 23.6% were controlling or losing weight, 32.2% were cutting down salt in diet, 23.5% were exercising, 13.1% were cutting down on alcohol intake and 8.5% quitted smoking (Table 2).

**Table 2:** Hypertension treatment categories.

Hypertension treatments	Number	Percentage
Taking prescribed medication	561	32.6
Controlling or losing weight	406	23.6
Cutting down salt in diet	554	32.2
Exercise to control hypertension	405	23.5
Cutting down on alcohol intake	225	13.1
Stopped smoking	147	8.5

### Systolic and diastolic blood pressure values

**Fig 1:**
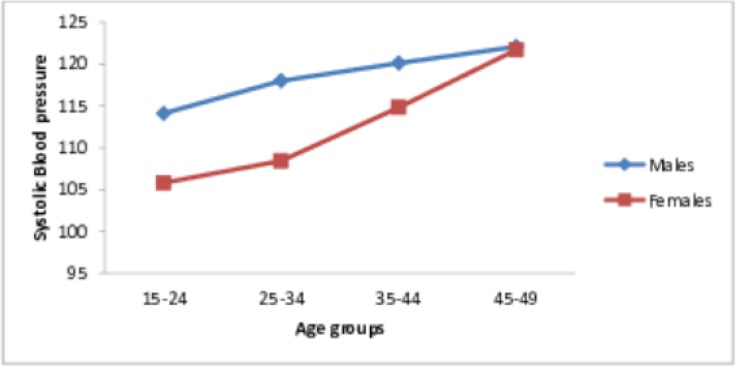
Distributions of the systolic blood pressure by age and sex.

**Fig 2:**
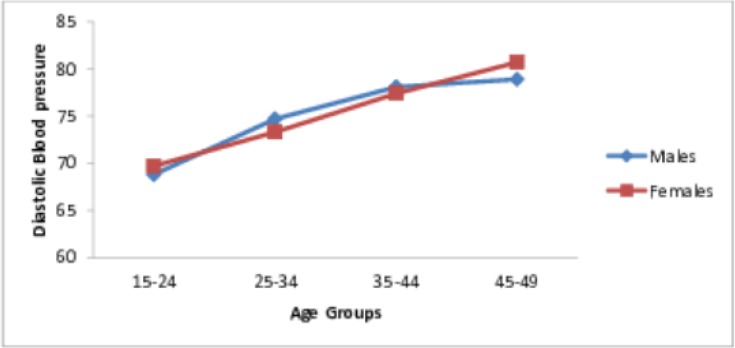
Distributions of the diastolic blood pressure by age and sex.

The mean systolic blood pressure (SBP) was 112.3 (±15.8) mmHg and it was significantly higher in men (117.3± 14.3) than in women (110.3 ± 15.9) (P <0.001). On the other hand, the mean diastolic blood pressure (DBP) was 73.7 (±11.3) mmHg and there was no significant difference between men (73.6 ± 11.2) and women (73.7 ± 11.4) (P >0.05). The distribution of SBP and DBP by sex and age groups, are shown in Figs 1 and 2. SBP was higher in men than in women in all age groups (Fig 1). In contrast, while DBP was higher among women than men in the youngest and oldest age groups (15–24 years and 45–49 years); it was higher for men from 25–44 years (Fig 2).

### Distribution of hypertension prevalence, awareness, treatment and control by socioeconomic, sociodemographic and lifestyle factors

Table 3 presents the Chi-Square results showing the variation in hypertension prevalence, awareness, treatment and control by sociodemographic, socioeconomic and lifestyle factors. Out of those who had hypertension, 45.6% (CI: 43.3–48.0) were aware of their hypertension status, 40.5% (CI: 38.2–42.8) were on treatment, and 23.8% (CI: 21.8–25.9) had their blood pressure controlled.

**Table 3:** Chi-Square tests showing the distribution in hypertension prevalence, awareness, treatment and control by background characteristics.

	^*$*^Prevalence (%)	^*Ȼ*^Awareness (%)	^*€*^Treatment (%)	^*£*^Control (%)
	N = 13,247	N = 1723	N = 1723	N = 1723
Overall	13.0 (12.4–13.6)	45.6 (43.3–48.0)	40.5 (38.2–42.8)	23.8 (21.8–25.9)
**Sex**				
Male	12.1	31.5***	88.4	53.1
Female	13.4	50.9	88.9	52.1
**Age**				
15–24	3.7***	38.0	80.9*	75.0***
25–34	11.1	45.1	85.9	61.8
35–44	21.8	46.2	90.1	47.4
45–49	32.9	48.5	92.4	42.9
**Marital status**				
Never married	5.2***	32.6***	82.7	66.7*
Currently married	16.6	47.9	89.3	51.0
Formerly married	24.3	48.7	90.5	49.2
**Level of education**				
No education	12.5	42.2	89.8	55.8
Primary/JHS	13.1	45.1	89.0	48.9
Secondary and above	13.2	49.9	87.6	57.2
**Occupational physical activities**				
Unemployed	6.7***	47.8***	86.5	58.3
Sedentary work	18.2	33.8	85.2	40.7
Moderate intense work	18.8	50.7	91.3	52.9
Vigorous intense work	11.3	39.4	85.7	50.0
**Wealth status**				
Poorest	7.4***	38.4**	81.9	13.6
Poorer	9.9	38.8	90.5	10.7
Middle	13.9	45.8	89.2	20.4
Richer	16.8	46.7	91.8	26.8
Richest	19.3	52.2	88.0	28.5
**Place of residence**				
Rural	9.6***	40.8**	89.4	54.5
Urban	16.6	48.6	87.6	47.9
**Region**				
Western	12.4***	39.0***	87.3*	42.3
Central	13.3	32.8	94.7	52.6
Greater Accra	19.4	54	81.9	47.7
Volta	16.1	49.4	89.8	45.5
Eastern	13.8	54.2	89.6	57.3
Ashanti	16.6	43.5	96.1	52.4
Brong Ahafo	11.4	35.8	93.1	55.2
Northern	9.2	52.9	84.7	61.1
Upper East	9.4	50	90.2	62.3
Upper West	7.6	39.2	83.9	54.8
**Ethnicity**				
Akan	14.9***	39.8***	91.3	50.5
Ga Adangbe	15.9	63.9	81.6	42.1
Ewe	16.2	51.6	89.3	53.4
Mole-Dagbani	9.7	47.9	85.8	56.8
Others	10.2	47.0	90.0	57.2
**Smoking Status**				
Non-smokers	13.0	46.0*	88.7	52.1
Smokers	12.5	25.7	100.0	66.7
**NHIS coverage**				
No	11.5***	35.0***	84.8*	51.0
Yes	13.9	51.1	90.2	52.8
**Visited to health facility in the last 6 months**				
No visit	11.8***	36.8***	85.7**	50.1
Hypertension related	91.8	95.5	96.5	58.8
Non-hypertension related	13.8	54.4	90.7	53.2
***Fruit consumption***	2.5	2.4	2.4	2.4
***Vegetable consumption***	2.7	2.7	2.7	2.7

*P < 0.05

** P < 0.01

***P < 0.001

^$^Reference group: participants not hypertensive

^Ȼ^Reference group: participants not aware of their hypertension

^€^Reference group: participants not treating their hypertension

^£^Reference group: participants who did not have their blood pressure controlled

The results in Table 3 showed that there was no significant difference in hypertension prevalence between males and females. While hypertension awareness was significantly higher for females compared to males (50.9% vs. 31.5%), hypertension treatment and control did not differ for both sexes. In terms of age, whereas hypertension prevalence, awareness and treatment increased with age, hypertension control reduced with age. Compared to those who had never been married, those who had ever been in a union had a higher proportion of hypertension prevalence, awareness and treatment; however, hypertension control was higher among those who had never been married, compared to their counterparts.

Hypertension prevalence, awareness, treatment and control did not differ by respondents’ levels of education. Further, those in level 1 occupation (i.e. Unemployed) had the lowest hypertension prevalence and the highest proportion of hypertension control. While hypertension prevalence was highest among those who were in level 3 occupation, hypertension awareness and treatment were lowest among those in level 2 occupation. Even though hypertension prevalence and awareness, increased with wealth status, hypertension treatment and control did not vary by wealth status.

In terms of place of residence, those who lived in urban areas had a higher hypertension prevalence, awareness and control; however, hypertension treatment did not differ by place of residence. Hypertension prevalence was highest in the Greater Accra region (19.4%), followed by the Ashanti region (16.6%) and lowest in the Upper West region (7.6%). With regard to hypertension awareness, the proportion varied from 32.8% in the Central region to 54.0% in the Greater Accra region. In addition, while hypertension treatment was highest in the Ashanti region (96.1%), it was lowest in the Greater Accra region (81.9%). Hypertension control was highest among Upper East region residents (62.3%) whereas Western region residents had the lowest hypertension control (42.3%).With regard to ethnicity, hypertension prevalence was highest among the Ewe (16.2%) and lowest among the Mole Dagbani (9.7%). While hypertension awareness was highest among the Ga-Adangbe (63.9%), those who were Akan had the lowest awareness rate (39.8%). Hypertension treatment and control, however, did not differ by ethnicity.

Further, the results showed that there were no significant differences in hypertension prevalence, treatment and control between smokers and non-smokers; however, hypertension awareness was significantly higher among non-smokers (46.0%) compared to smokers (25.7%). Also, those who were on National Health Insurance Scheme (NHIS) had a higher hypertension prevalence (13.9% vs. 11.5%), awareness (51.1% vs 35.0%) and treatment (90.2% vs. 84.8%) compared to those who were not covered by NHIS. Hypertension control did not differ by NHIS coverage. Further, hypertension prevalence, awareness, and treatment were higher among those who visited health facilities in the last six months for hypertension related issues compared to their counterparts who visited for other reasons and those who did not visit at all; however, there was no difference in hypertension control between these three groups. There was no statistically significant difference between fruit and vegetable consumptions and hypertension prevalence, awareness, treatment and control.

### Correlates of hypertension prevalence, awareness, treatment and control

The correlates of hypertension prevalence, awareness, treatment and control are shown in Table 4. The correlates of hypertension prevalence include: advancement in age, being married, being formerly married, higher wealth status, living in urban area, living in Western and Upper West Regions, belonging to Ewe and other ethnic group, being covered by NHIS and visit to a health facility for hypertension related issues 6 months prior to the survey.

**Table 4:** Correlates of hypertension prevalence, awareness, treatment and control.

	Prevalence		Awareness		Treatment		Control	
Characteristics	Odds Ratio	95% CI	Odds Ratio	95% CI	Odds Ratio	95% CI	Odds Ratio	95% CI
**Sex**								
Female (RC)	1.00		1.00		1.00		1.00	
Male	1.03	0.86–1.23	0.52**	0.36–0.76	1.40	0.54–3.62	1.01	0.60–1.72
**Age**								
15–24 (RC)	1.00		1.00		1.00		1.00	
25–34	2.53***	1.71–3.75	1.39	0.53–3.62	1.40	0.25–7.92	0.70	0.13–3.71
35–44 45–49	3.50***	2.39–5.14	1.23	0.53–3.62	1.96	0.33–11.77	0.33	0.07–1.59
**Marital status**								
Never married (RC)	1.00		1.00		1.00		1.00	
Currently married	1.18	0.94–1.48	1.79*	1.14–2.83	1.15	0.42–3.17	1.22	0.58–2.57
Formerly married	1.36*	1.02–1.83	1.61	0.94–2.77	1.56	0.46–5.33	1.10	0.45–2.70
**Level of education**								
No education (RC)	1.00		1.00		1.00		1.00	
Primary/JHS	1.16	0.92–1.44	1.43	0.96–2.15	1.90	0.70–5.12	1.05	0.58–1.88
Secondary and above	1.13	0.91–1.40	1.25	0.82–1.89	0.83	0.34–2.05	0.76	0.41–1.40
**Occupational physical activities**								
Sedentary work (RC)	1.00		1.00		1.00		1.00	
Unemployed	1.01	0.71–1.45	1.72	0.76–3.86	1.39	0.25–7.88	1.46	0.46–4.69
Moderate intense work	1.11	0.79–1.37	1.52	0.76–3.03	1.94	0.40–9.37	2.11	0.70–6.42
Vigorous intense work	0.90	0.63–1.28	1.50	0.71–3.15	1.36	0.30–6.29	1.94	0.64–5.88
**Wealth status**								
Poorest (RC)	1.00		1.00		1.00		1.00	
Poorer	0.99	0.77–1.29	1.03	0.58–1.84	1.63	0.54–4.88	0.65	0.32–1.30
Middle	1.35*	1.04–1.76	1.23	0.74–2.06	2.40	0.79–7.30	0.80	0.37–1.73
Richer	1.65**	1.21–2.25	1.63	0.89–2.98	2.58	0.84–7.94	0.90	0.40–2.05
Richest	1.75**	1.25–2.46	1.72	0.88–3.35	2.19	0.57–8.38	0.66	0.27–1.62
**Place of residence**								
Rural (RC)	1.00		1.00		1.00		1.00	
Urban	1.23*	1.04–1.47	0.79	0.57–1.09	1.12	0.49–2.53	1.63	0.94–2.82
**Region**								
Greater Accra (RC)	1.00		1.00		1.00		1.00	
Western	0.75*	0.58–0.97	0.74	0.43–1.26	1.63	0.57–4.63	0.91	0.43–1.93
Central	0.74	0.63–1.02	0.67	0.40–1.14	3.63	0.86–15.34	1.42	0.61–3.31
Volta	1.06	0.75–1.50	0.82	0.46–1.46	2.81	0.83–9.47	0.84	0.35–2.01
Eastern	0.86	0.66–1.14	1.13	0.63–2.01	1.48	0.55–3.99	2.02*	1.09–3.74
Ashanti	0.98	0.76–1.27	0.84	0.48–1.45	5.97*	1.44–24.77	1.27	0.70–2.30
Brong Ahafo	0.84	0.64–1.11	0.75	0.42–1.33	4.02*	1.20–13.49	1.73	0.80–3.72
Northern	0.85	0.59–1.23	1.12	0.55–2.28	1.74	0.33–9.12	2.09	0.74–5.92
Upper East	0.89	0.59–1.35	1.03	0.46–2.32	6.86*	1.53–30.84	3.78	1.35–10.57
Upper West	0.67*	0.45–0.97	0.67	0.31–1.47	3.99	0.76–20.88	1.99	0.66–5.96
**Ethnicity**								
Ga Adangbe (RC)	1.00		1.00		1.00		1.00	
Akan	1.29	0.99–1.71	0.45**	0.26–0.76	1.44	0.61–3.42	1.00	0.49–2.02
Ewe	1.42	1.00–1.92	0.71	0.39–1.28	1.35	0.41–4.50	1.93	0.82–4.54
Mole-Dagbani	1.04	0.74–1.46	0.56	0.25–1.24	0.49	0.14–1.66	0.68	0.25–1.82
Others	1.70*	1.12–2.58	0.40*	0.18–0.94	0.71	0.10–5.35	1.49	0.31–7.25
**Smoking**								
Non-smokers (RC)	1.00		1.00		-	-	1.00	
Smokers	1.03	0.62–1.72	0.58	0.22–1.54	-	-	2.98	0.62–14.23
**NHIS coverage**								
No	1.00		1.00		1.00		1.00	
Yes	1.20*	1.04–1.38	1.60**	1.21–2.11	1.10	0.58–2.11	0.94	0.59–1.51
**Visit to health facility in the last 6 months**								
No visit (RC)	1.00		1.00		1.00		1.00	
Hypertension related	40.85***	19.08–87.48	30.53***	10.42–89.52	3.66	0.99–13.53	1.64	0.88–3.06
Non-hypertension related	1.01	0.87–1.17	1.50**	1.12–2.00	1.79	0.96–3.33	1.45	0.95–2.22
***Fruit consumption***	0.98	0.96–1.02	0.95*	0.90–0.99	1.04	0.93–1.16	1.01	0.93–1.10
***Vegetable consumption***	0.99	0.96–1.02	0.97	0.91–1.02	1.01	0.91–1.12	0.95	0.88–1.03

*P < 0.05

** P < 0.01

***P < 0.001

CI- Confidence interval

Further, being currently married, having a secondary education and higher, being covered by NHIS and visit to a health facility in the last 6 months prior to the survey for hypertension and non-hypertension related issues increased the odds of hypertension awareness. However, the odds of hypertension awareness was lower for males compared to females [Odds Ratio (OR) = 0.54, p < 0.001]. Those who were Akan significantly had lower odds of hypertension awareness compared to those who were Ga Adangbe. An increase in the number of days that fruit was consumed reduced the odds of hypertension awareness [Odds Ratio (OR) = 0.95, p < 0.05]. The results showed that hypertension awareness increased with level of wealth status, although this was not statistically significant.

The predictors of hypertension treatment included region of residence and health facility visit. Particularly, the odds of hypertension treatment was higher among those in Ashanti and Brong Ahafo regions, compared to those in Greater Accra region. Also, people who visited the health facility in the last 6 months were 2.57 times more likely to treat their hypertension compared to those who did not visit the health facility within this period.

Further, our results showed that age and region of residence were predictors of hypertension control. Compared to those who were 15–24 years, those who were 25–34 years, 35–44 years and 45–49 years significantly had lower odds of hypertension control (OR = 0.41, 0.19, and 0.16, respectively). Hypertension control was significantly higher among those in Eastern and Upper East regions, compared to those in Greater Accra region.

## Discussion

The discussion is organised around two main themes: 1) hypertension prevalence; and 2) hypertension awareness treatment and control.

### Hypertension prevalence

This is the first national study to examine hypertension prevalence, awareness, treatment and control in this population. This study showed that hypertension prevalence among Ghanaians aged 15–49 years was 13.1%, and the correlates were advancement in age, being formerly married, higher wealth status, living in urban area, living in Western, Central, and Brong Ahafo regions, being covered by NHIS and visit to health facility over the last 6 months prior to the survey.

The findings showed that hypertension prevalence ranged from 2.4% among those who were 15–19 years to 32.9% among those who were 45–49 years. Clearly, hypertension prevalence increased with advancement in age and this aligns with what previous studies have shown [23,28,29]. Hypertension prevalence increases with age because of: changes in arterial and arteriolar stiffness, decreased baroreceptor sensitivity, increased responsiveness to sympathetic nervous system stimuli, altered renal and sodium metabolism and altered renin-aldosterone relationship [30] chronic, low-grade inflammation and increased cellular oxidative stress [31]. Even though the hypertension prevalence reported in this study was relatively low, slightly more than one-fifth (22%) were pre-hypertensives. This indicates a high proportion of people that are at high risk of hypertension. It is important to develop primary intervention strategies that target both the high (pre-hypertensives) and the low risk (normotensives) groups.

Further, hypertension prevalence was higher among those who were formerly married and this is similar to what other studies have shown [32]. Studies have shown that people who are divorced, widowed or separated have poorer cardiovascular health outcomes compared to married individuals. Particularly, marriage has been seen to be protective against cardiovascular outcomes [32]. Explanations for high rate of hypertension prevalence among those who were formerly married may be that they probably had low access to income and health care facilities. Research has shown that people in marriage have better quality of health due to better access to income and health insurance, and higher level of social support which helps to prevent them from engaging in risky behaviours [33].

Hypertension was also found to be more prevalent among those with higher wealth status and those living in urban areas. Research has shown that socioeconomic status may shape the lifestyles of individuals and which may predispose them to hypertension [23]. This is contrary to a meta-analysis, which showed that low socioeconomic status is associated with higher hypertension [34]. A plausible explanation for this may be due to the structural setting in Ghana. In Ghana, many individuals with high socioeconomic status and living in urban areas face tremendous psychosocial stress due to various hassles, deadlines, demands, traffic situation, and frustrations that they experience on a daily basis This is plausible because studies have shown that psychosocial stress is a major risk factor for hypertension and other cardiovascular diseases [35]. It may be interesting for future studies to examine the impact of psychosocial on hypertension prevalence in urban areas in Ghana.

### Hypertension awareness, treatment and control

Hypertension awareness, treatment and control are vital for prevention of CVDs and complications [36]. In the present study, awareness of hypertension was 46%. Compared to a study in an urban poor community in Accra, the awareness of hypertension was high in this study (7.4%) [23]. However, one must not be complacent as the 54% who are unaware of their status can be at risk of stroke and other CVDs [37]. We found that the awareness of hypertension was associated with gender, marital status, level of education, ethnicity coverage of NHIS and health facility visit in the last six months prior to the survey. These findings are consistent with those from other studies [37–40]. The gender differences in awareness of hypertension status can be explained by women’s health seeking behaviours. For instance, the majority of Ghanaian women have registered with the NHIS compared to men [41–44]. On the other hand, studies have reported men’s unwillingness to report and seek medical attention; this behaviour could account for low awareness of a disease, especially with an asymptomatic condition like hypertension [45,46].

More than two-fifth (40.5%) of the hypertensive patients in this study were on treatment. Region of residence was the only significant variable related to treatment of hypertension. It is interesting that patients in the relatively deprived regions (i.e. Ashanti, Brong Ahafo and Upper East) had higher levels of treatment compared to those in the Greater Accra region. Plausible explanations can be the influx of civil society agencies providing health services in these communities [44,47]. Also, it could be that individuals in these areas are not under the pressure of the “Accra life” and have the opportunity to visit health facilities. Hospitals in the Greater Accra region are mostly crowded and with long waiting periods which might discourage patients from seeking treatment [48,49].

Poor hypertension control, despite a relatively high rate of awareness, has been observed by our study and several other studies as well [6, 12]. Control of hypertension among the participants was 23.8% in this study. This level of control is relatively high compared to what is found in other studies in Ghana and Uganda [50,51] but lower than the one reported in a hospital study in Uganda [51]. Poor control of hypertension may be attributed to both health system factors and patient factors. The lack of anti-hypertensive medication, long distance to hospitals, high cost of drugs, inadequate counselling, and lack of appropriate knowledge, skill and resources to tackle hypertension are some of the health system related factors associated with poor hypertension control [12]. A key component of patient factors is that, hypertension is asymptomatic (does not present symptoms), and usually at variance with the conception of disease of most people in Ghana [52,53]. For example, in a context where disease is recognised or identified by symptoms, unavailability of symptoms makes it difficult for people to accept their condition of ill health and to commence appropriate treatment actions [54]. Further, research shows even when people accept their conditions, lifelong management which includes long term pharmacotherapy and significant lifestyle changes can be challenging for low income people living with chronic diseases [55]. Although all the classes of anti-hypertensive medications are commonly available for people living with hypertension in Ghana; however, not all the drugs are listed on the NHIS. Even when they are listed on the NHIS drug list for hypertension, a lot of times, patients cannot access them because of frequent drug stock-outs in many medical stores in the country. In such instances, patients have to buy these drugs out- of-pocket; this increases the cost of health care and may consequently result in inadequate treatment and poor hypertension control.

Age was the only significant predictor of hypertension control in this study. Those who were 35–49 years particularly had poorer hypertension control compared to those who were 15–19 years. A plausible explanation is majority of those who were 35–49 years were working, and probably have more dependants; hence, they may not have enough time to take care of themselves or manage their hypertension. On the other hand, those who were 15–19 years are more or less dependants and perhaps received lots of support for managing their hypertension.

It is surprising that a visit to the hospital was not significantly associated with hypertension control even though according to the treatment guidelines of the Ghana Health Services (GHS), hypertension patients have a quarterly appointment to health facilities for review and treatment [26]. Our finding clearly shows that there is a gap between treatment guidelines and the reality on the ground. Based on this finding, we recommend that intervention strategies should be directed at encouraging people living with hypertension in Ghana to make frequent visits to health facilities for regular medical check-ups. Coupled with this, there is a need to equip the health facilities with necessary tools that can enhance hypertension management and control. Further, there is a need for Ghana Health Service to explore the possibility of reducing the quarterly hospital appointment of hypertensive patients to a more frequent period.

### Limitations

This study is not without limitations. The study was biased towards women as the majority of the respondents were women. During the survey, blood pressure measurements were taken for all the women who consented in the selected households, whereas blood pressure measurements were taken for only half of the men in the selected households. The DHS sampling process is designed to select more women than men [24]. This study showed that hypertension awareness was higher for women compared to men and this may be due to the oversampling of women. In order to address this limitation in the future, we recommend that in subsequent GDHS, Ghana Statistical Service should collect information on blood pressure measurements for all eligible males in order to see the true picture of hypertension awareness in this population.

Further, it must also be noted that the hypertension prevalence among the sample may have been underestimated, because the blood pressure measurements were collected only at a single visit and were not temperature adjusted. This is one of the limitations of cross sectional data collection as this kind of data cannot be used to estimate prevalence of hypertension over time [56]. Further, the 2014 GDHS data did not allow in-depth examination of the types of treatment adopted by the participants. Qualitative studies have shown that people living with chronic noncommunicable diseases (NCDs) use combination of biomedicine, ethnomedicine, and faith healing in managing their condition [18,57,58]. Introducing questions that examine health seeking behaviours into national surveys will provide a great tool for surveillance of NCDs.

## Conclusion

Although, hypertension is a rising problem in Ghana, knowledge of the prevalence, awareness, treatment and control at the national level is limited. This study used a national level data to examine the prevalence and determinants of hypertension, awareness, treatment and control. This study provides an empirical data on hypertension prevalence, awareness, treatment and control among Ghanaians aged 15–49; however, there is limitation to the generalizability of the results because of the bias due to limited male data. Nevertheless, the findings provide vital information for health service planning and provision of medical services. Secondly, this study shows the factors that are important for developing intervention strategies among people aged 15–49 years in Ghana. This context specific knowledge is important for examining the individuals who are at risk and for providing targeted services to such persons. Thirdly, the study showed that some of the determining factors such as age, gender, marital status are similar to other cultures; therefore, existing interventions from those cultures could be adapted in addressing hypertension prevalence, awareness, treatment and control in Ghana.

## Supporting information

S1 Table Multicollinearity tests. (DOCX)
