# Supplementary material for: Hypertension prevalence, awareness, treatment and control in Ghanaian population: Evidence from the Ghana demographic and health survey
Source: PLoS One. 2018 Nov 7;13(11):e0205985. doi: 10.1371/journal.pone.0205985 (PMC6221286; doi:10.1371/journal.pone.0205985)
Supplement: S1 Table — (DOCX) [file pone.0205985.s001.docx]

**S1 Table. Multicollinearity tests.**

| **Variable** | **VIF** | **1/VIF** |
| --- | --- | --- |
| **Sex** |  |  |
| Male | 1.33 | 0.751146 |
| Female | - | - |
| **Age** |  |  |
| 15-24 | - | - |
| 25-34 | 2.14 | 0.468164 |
| 35-44 | 2.47 | 0.402933 |
| 45-49 | 1.75 | 0.571408 |
| **Marital status** |  |  |
| Never married | - | - |
| Currently married | 2.44 | 0.409225 |
| Formerly married | 1.60 | 0.623575 |
| **Level of education** | |  |
| No education | - | - |
| Primary/JHS | 2.31 | 0.432649 |
| Secondary and above | 2.75 | 0.363447 |
| **Occupational physical activities** | |  |
| Unemployed | 6.95 | 0.143834 |
| Sedentary work | - | - |
| Moderate intense work | 7.94 | 0.125971 |
| Vigorous intense work | 8.68 | 0.115273 |
| **Wealth status** |  |  |
| Poorest | - | - |
| Poorer | 1.85 | 0.541183 |
| Middle | 2.34 | 0.42774 |
| Richer | 3.04 | 0.329482 |
| Richest | 3.84 | 0.260609 |
| **Place of residence** | |  |
| Rural | - | - |
| Urban | 1.96 | 0.509999 |
| **Region** |  |  |
| Western | 2.26 | 0.442533 |
| Central | 2.14 | 0.46719 |
| Greater Accra | - | - |
| Volta | 2.48 | 0.402933 |
| Eastern | 1.93 | 0.517493 |
| Ashanti | 2.10 | 0.476565 |
| Brong Ahafo | 2.37 | 0.421617 |
| Northern | 3.09 | 0.323663 |
| Upper East | 3.02 | 0.331113 |
| Upper West | 2.63 | 0.380446 |
| **Ethnicity** |  |  |
| Akan | 5.91 | 0.169277 |
| Ga-Adangbe | - | - |
| Ewe | 3.52 | 0.283994 |
| Mole-Dagbani | 6.12 | 0.163322 |
| Others | 4.50 | 0.222165 |
| **Smoking** |  |  |
| Non-smokers | - | - |
| Smokers | 1.07 | 0.933845 |
| **NHIS coverage** |  |  |
| No | - | - |
| Yes | 1.17 | 0.85745 |
| **Visit to health facility in the last 6 months** | |  |
| No visit (RC) | - | - |
| Hypertension related | 1.03 | 0.97432 |
| Non-hypertension related | 1.16 | 0.858814 |
| ***Fruit consumption*** | 1.14 | 0.873948 |
| ***Vegetable consumption*** | 1.13 | 0.887394 |

***VIF- Variance inflation factor; 1/VIF- Tolerance***
